# Social Comparison Orientation and Social Adaptation Among Young Chinese Adolescents: The Mediating Role of Academic Self-Concept

**DOI:** 10.3389/fpsyg.2018.01067

**Published:** 2018-06-27

**Authors:** Hualing Miao, Zhenxing Li, Yingkai Yang, Cheng Guo

**Affiliations:** ^1^The Lab of Mental Health and Social Adaptation, Faculty of Psychology, Southwest University, Chongqing, China; ^2^Research Center of Mental Health Education, Southwest University, Chongqing, China; ^3^School of Education, Huaibei Normal University, Huaibei, China

**Keywords:** social comparison orientation, social adaptation, academic self-concept, young adolescents, Chinese

## Abstract

This study aimed to investigate the relationship among social comparison orientation, academic self-concept (ASC), and social adaptation. A total of 1658 Chinese adolescents (48.88% male; aged 14–18 years, *M*_age_ = 16.01 ± 0.86 years) voluntarily participated in this study and completed questionnaires. Structural equation modeling (SEM) was performed to test the theory-driven model. The results showed that the relationship between comparison of opinion and social adaptation was mediated by ASC but that ASC did not play a mediating role between comparison of ability and social adaptation. These findings indicated that ASC could be one mechanism explaining the link between adolescents’ social comparison orientation and social adaptation. Furthermore, it is possible to intervene in their social comparison orientation and ASC to improve adolescents’ social adaptation.

## Introduction

Social adaptation is the degree to which individuals engage in competent social activities or adapt to the immediate social environment (Crick and Dodge, 1994). Currently, social adaptation does not have a clear and operational definition. From the functionalistic perspective, social adaptation could be divided into positive social adaptation (PSA) and negative social adaptation (NSA). PSA involves behaviors through which individuals meet their needs for survival, development, or social norms, and it is related to their happiness, strength, and growth. It includes four dimensions: self-affirmation, pro-social tendency, acting sufficiency, and active coping. NSA involves behaviors that are not consistent with individual self-satisfaction or social responsibility or that are not conducive to individuals’ survival, development, or growth. It includes four dimensions: self-trouble, social alienation, violations, and social withdrawal (Zou et al., 2012). Previous studies have shown that social adaptation was correlated to specific internal factors such as personality traits (Huntsinger and Jose, 2006) and self-concept (Buhs, 2005; Romera et al., 2016; Lim and Lee, 2017; Suszek et al., 2018). One highly relevant personality trait would be social comparison orientation (Yang, 2016), which refers to the tendency to compare oneself with others (Gibbons and Buunk, 1999). Previous studies found that social comparison orientation was strongly associated with PSA (e.g., life satisfaction) and NSA (e.g., depression, low happiness) (Gibbons and Buunk, 1999; Alderson and Katz-Gerro, 2016). Another interesting factor influencing social adaptation is academic self-concept (ASC). In a multifaceted hierarchical model, self-concept is generally divided into academic and non-ASC (Shavelson et al., 1976). ASC is an evaluative self-perception formed through the students’ experience and interpretation of the school environment (Shavelson et al., 1976; Marsh and Craven, 1997). For adolescents, as school represents a major life domain (Huebner, 1994), ASC is particularly critical for their adaptation (Wouters et al., 2011; Rodríguez et al., 2012; Dadarigashti et al., 2016).

Social adaptation is a well-known indicator of health, and it is essential to improve our understanding of the relationship among social comparison orientation, ASC, and social adaptation. Some studies have reported that social comparison orientation was directly or indirectly correlated to many psychological outcomes (Yang, 2016; Kim et al., 2017). Moreover, social comparison theory, the external frame of the reference model and the big-fish-little-pond effect hold that social comparison is the basis for the formation of ASC (Festinger, 1954; Marsh, 1984; Möller and Marsh, 2013). Therefore, this study aimed to explore the relationship between social comparison orientation and social adaptation, as well as the mediating role of ASC.

### Direct Relationship Between Social Comparison Orientation and Social Adaptation

A central tenet of social comparison theory is that individuals compare themselves with others when they are uncertain about their opinions and abilities, especially in the absence of objective standards (Festinger, 1954). In our everyday life, social comparison is universal and valuable. From an evolutionary perspective, Gilbert et al. (1995) proposed that the need to compare oneself with others is very old in terms of phylogenetic development, biologically very powerful, and recognizable in many species, given the adaptive value of adequately sizing up one’s competitors. Social comparison theory also suggests that social comparison has an important influence on human behavior and psychology (Festinger, 1954). Based on previous studies, it may be established that social comparison is closely related to social adaptation. On the one hand, individuals with high social comparison frequencies were uncertain about various aspects of themselves and needed to use information with regard to other people for self-evaluation (Festinger, 1954); in other words, these individuals were more vulnerable to comparison information (Buunk et al., 2001). High social comparison frequencies may be correlated to undesirable results, such as feelings of academic inferiority (Burleson et al., 2005), low happiness (Alderson and Katz-Gerro, 2016), poor self-perception, low self-esteem, and social anxiety (Gibbons and Buunk, 1999; Vogel et al., 2015). On the other hand, individuals with high social comparison frequencies tended to accept others’ views or ideas and spent more time in the process of attention and cognitive construction, which could help individuals use proactive strategies (Affleck and Tennen, 1991; Gibbons and Buunk, 1999). In general, social comparison was correlated to positive and negative outcomes, which in some ways are manifestations of social adaptation. Moreover, social comparison theory proposed that both a person’s cognition (opinions and beliefs) about the situation in which he/she exists and a person’s appraisals of what he/she is capable of doing (evaluation of own abilities) will have a bearing on his/her behavior (Festinger, 1954). Thus, social comparison was directly related to social adaptation (Yang, 2016).

Moreover, it is necessary to consider why this duality occurred in high social comparison frequencies. Gibbons and Buunk (1999) used the term social comparison orientation to explain individual differences in social comparison, and it includes two dimensions: comparison of ability and comparison of opinion. Comparison of ability means that an individual tends to compare his/her abilities and achievements with those of others, while comparison of opinion means that an individual tends to compare his/her ideas and beliefs with those of others. Regarding ability, the primary question is “How am I doing?”; for opinion, the question is “What should I think or feel?” (Gibbons and Buunk, 1999). There were differences between the two orientations: individuals with low self-esteem, high depression, and high neuroticism tended to engage in ability-based comparisons but were less likely to participate in opinion-based comparisons than those with opposite levels of self-esteem, depression, and neuroticism (Gibbons and Buunk, 1999). Similarly, a previous study suggested that comparison of ability was positively associated with personal relative deprivation but that comparison of opinion was negatively associated with personal relative deprivation (Callan et al., 2015). As Smith et al. (2012) outlined, personal relative deprivation is characterized by feelings of anger, resentment, and frustration in response to disadvantaged social comparisons with relevant others. Usually, the affective component (e.g., depression, resentment, or loneliness) is treated as an indirect measurement index of social adaptation (Watson and Friend, 1969; Kahle, 1984). Therefore, it could be concluded that social comparison orientation was directly correlated to social adaptation, with comparison of ability and comparison of opinion playing distinct roles.

Furthermore, it is particularly important to explore why these two kinds of social comparison orientations were in different directions. Suls and Miller (1979) suggested that there was a “non-social restraint” in the process of comparison of ability, and that this did not exist in the comparison of opinion. Social comparison theory proposes that if a person changes his/her mind about something and abandons one belief in favor of another, there is no further difficulty regarding consummating the change. However, even if a person is convinced that he/she should be able to run faster or should be more intelligent than he/she currently can or is and even if he/she is highly motivated to improve his/her ability in some respects, there are great difficulties regarding consummating such a change (Festinger, 1954). According to non-social restraints, individuals can completely change or modify their opinions when they discover that their point of view is very different from that of others; however, they cannot completely change their abilities (Suls and Miller, 1979). Consequently, non-social restraints may be one reason for the inverse relationship of social comparison orientations and social adaptation. Based on the above research, we can postulate that comparison of opinion is positively correlated to social adaptation, whereas comparison of ability is negatively correlated to it.

### Indirect Relationship Between Social Comparison Orientation and Social Adaptation

Notably, the relationship between social comparison orientation and social adaptation may include direct and indirect correlations (Yang, 2016; Kim et al., 2017). In this study, we attempted to explain the inconsistent relationship between social comparison orientation and social adaptation by providing an explanatory mechanism. Therefore, we explored a motivational factor repeatedly found to be associated with adaptation, namely, ASC (Wouters et al., 2011; Rodríguez et al., 2012; Dadarigashti et al., 2016). ASC refers to a student’s attitudes, feelings, and perceptions regarding his/her academic abilities or skills (Lent et al., 1997). The link between ASC and individual adaptation has been well established in empirical research. For instance, some studies indicated that ASC was associated with academic adaptation, such as academic emotion (Goetz et al., 2006), test anxiety level (Lohbeck et al., 2016), and academic achievement (Khalaila, 2015; Albert and Dahling, 2016). However, the strength of the influence and contribution of ASC to adolescents’ non-academic adaptation (e.g., social adaptation) is uncertain. In this study, we aimed to examine the direct relationship between ASC and social adaptation.

On the other hand, some previous studies showed that social comparison was associated with adolescents’ ASC (Marsh, 1984; Möller and Marsh, 2013). Meanwhile, social comparison has also been explored as a variable in the consideration of various facets of ASC. The external frame of reference model by Marsh (e.g., Marsh, 1990a) assumes that students’ ASC is formed through comparing their performance with that of others. The big-fish-little-pond effect describes the fact that students in high-achieving groups develop lower self-concepts than equally capable students in low-achieving environments (Marsh, 1984; Marsh and Parker, 1984; Seaton et al., 2010). These two theories and social comparison theory have already inferred that social comparison is a key prerequisite for ASC (Festinger, 1954; Marsh, 1984; Möller and Marsh, 2013).

As a motivational factor, the role of ASC as a mediator has often been explored in research, and some empirical studies have provided evidence for it. For instance, Guay et al. (2010) found that ASC mediated the relationship between autonomous academic motivation and achievement. Another study found that ASC mediated the relationship between ethnic identity and grade point average (Cokley and Chapman, 2008). Furthermore, other studies also found that ASC played a mediating role in the relationship between academic achievement and psychological outcomes such as academic interest (Trautwein et al., 2006) and professional ambition, based on the big-fish-little-pond effect (Nagengast and Marsh, 2012). Based on the above studies, the relationship among comparison of ability, comparison of opinion, ASC, and social adaptation is complex and intercorrelated. In this study, we examined whether ASC could be a mechanism explaining the indirect relationship between adolescents’ social comparison orientation and their social adaptation.

### Current Study

Learning about oneself, consciously or unconsciously, through social comparison is ubiquitous (Corcoran et al., 2011). The finding that social comparison is closely related to public and private self-consciousness is evidence supporting this claim (Gibbons and Buunk, 1999; Neff and Vonk, 2009). Local research in China reported that vocational high school students in grades 1 and 2 experienced a rapid increase in self-consciousness (Han and Wei, 1985) and did not understand themselves clearly. Therefore, this heightened uncertainty may drive individuals to engage more in social comparison than otherwise (Gibbons and Buunk, 1999). At the same time, vocational students face numerous problems and considerable pressure (De Vries et al., 1994). For example, Chinese vocational students are faced with interpersonal relationship problems, the unruly behavior of peers, poor self-adaptation, and environmental stress (Shi, 2005), which are not conducive to effective social adaptation or mental health. In this context, it is important to investigate Chinese vocational students’ social comparison orientation and social adaptation. Therefore, the aim of this study was to explore the cross-sectional associations among social comparison orientation, ASC, and social adaptation in young Chinese adolescents. A clarification of the relationships among these variables may be useful in the implementation of specific prevention strategies for improving adolescents’ social adaptation.

**FIGURE 1:**
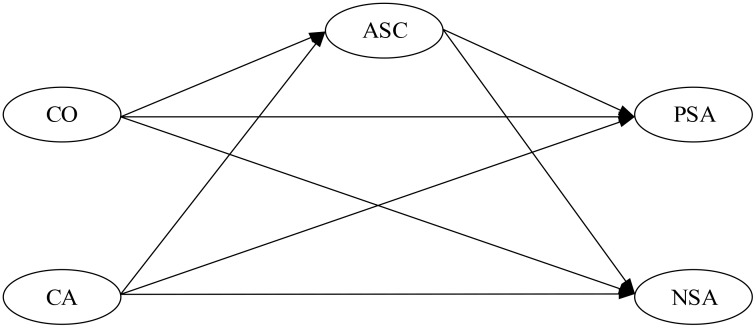
Hypothetical model. CO, comparison of opinion; CA, comparison of ability; ASC, academic self-concept; PSA, positive social adaptation; NSA, negative social adaptation.

Based on the above literature review and theories, first, we hypothesized that social comparison orientation was directly correlated to social adaptation and that comparison of ability was negatively correlated to PSA and positively correlated to NSA. Moreover, comparison of opinion was positively correlated to PSA and negatively correlated to NSA (H1). Second, comparison of ability was negatively correlated to ASC, and comparison of opinion was positively correlated to ASC (H2). Third, ASC was positively correlated to PSA and negatively correlated to NSA (H3). Finally, social comparison orientation was indirectly correlated to social adaptation, and ASC would mediate the relationship between social comparison orientation and social adaptation (H4). Based on the hypotheses above, the hypothetical model of the current study is shown in **Figure 1**.

## Materials and Methods

### Participants and Procedure

Young adolescents (*N* = 1658) from two vocational high schools in southwest China voluntarily participated in this study. The mean age of the participants was 16.01 years (*SD* = 0.86). A total of 1080 participants were in the 10th grade, 567 were in the 11th grade, and 11 participants did not report their grade. A total of 808 participants were boys, 845 participants were girls, and 5 participants did not report their gender. A total of 693 participants lived in urban households, 956 participants lived in rural households, and 9 students did not report their household type. In addition, all participants were fluent in Mandarin.

### Ethics Statement

This study was carried out in accordance with the recommendations of the ethics committee for psychological research at our university. All subjects gave written informed consent in accordance with the Declaration of Helsinki. The protocol was approved by the ethics committee of Southwest University.

Written consent was first obtained from the school administrators and students’ parents; subsequently, willing participants provided oral consent, and trained research assistants explained the guidelines for completing the questionnaires and assured the students that their participation was anonymous and voluntary. After completing the questionnaires, each participant was thanked verbally. The data were collected during a 40-min class.

### Measures

#### Iowa–Netherlands Comparison Orientation Measure

The Iowa–Netherlands Comparison Orientation Measure was used to measure social comparison orientation (Gibbons and Buunk, 1999). This self-report scale has previously been revised by Chinese scholars; the Chinese version was then determined to have acceptable reliability and validity, and has been applied in related psychological research areas (Wang et al., 2006). Comparison of opinion was measured with seven items, such as “I often like to talk with others about mutual opinions and experiences”; comparison of ability was measured with four items, such as “I often compare how I am doing socially (e.g., social skills, popularity) with other people.” The items were rated on a 5-point Likert scale ranging from 1 (*disagree strongly*) to 5 (*agree strongly*), with higher scores indicating higher levels of social comparison orientation. Two items (4 and 5) were reverse scored. In the current study, Cronbach’s α for social comparison orientation, comparison of ability, and comparison of opinion was 0.69, 0.66, and 0.64, respectively.

#### General Academic Self-Concept Subscale

We selected the general ASC subscale from the Self-description Questionnaire II, developed by Marsh (1990b) and revised by Chinese scholars (Chen and Cui, 1997). The revised Chinese version has acceptable reliability and validity and has been applied in related psychological research areas (Chen et al., 1997; Xu et al., 2008; Chen and Shi, 2016). This self-report subscale consists of 10 items rated on a 6-point Likert scale ranging from 1 (*totally unsuitable*) to 6 (*totally suitable*), with higher scores indicating better ASC. Five items (2, 4, 6, 8, and 10) are reverse scored. An example item is “I learn things quickly in most school subjects.” In this study, Cronbach’s α for the subscale was 0.82.

#### Adolescent Social Adaptation Assessment Scale

We used the Adolescent Social Adaptation Assessment Scale (ASAAS), developed by Zou et al. (2012), to measure the participants’ social adaptation. In previous studies, the ASAAS was proven to be reliable and valid in China (Liu et al., 2012; Yang et al., 2012). This scale comprises 50 items in two subscales: PSA (27 items; α = 0.92) and NSA (23 items; α = 0.89). The PSA subscale is further divided into lower order subscales: self-affirmation (eight items; α = 0.85), pro-social tendency (seven items; α = 0.82), acting sufficiency (six items; α = 0.78), and active coping (six items; α = 0.83). The NSA subscale is also further divided into lower-order subscales: self-trouble (eight items; α = 0.88), social alienation (five items; α = 0.66), violations (five items; α = 0.90), and social withdrawal (five items; α = 0.76). All items were rated on a 5-point Likert scale ranging from 1 (*completely unsuitable*) to 5 (*completely suitable*), and items 41 and 43 are reverse scored. Examples of items for the subscales are as follows: “I can face the difficulties of life and study” (PSA) and “I often feel helpless when dealing with problems in life” (NSA).

### Analytic Strategy

First, SPSS 21.0 (IBM Corp., Armonk, NY, United States) was used to calculate descriptive statistics and conduct correlation analyses and *t*-tests. Next, Mplus 7.0 (Muthén et al., 2012) was used to test the hypothesized models. Furthermore, a confirmatory factor analysis (CFA) was performed to test the measurement model of the constructs, and structural equation modeling (SEM) was conducted to test the mediating role of ASC in the relationship between social comparison orientation and social adaptation. CFA and SEM were conducted using the mean-adjusted maximum likelihood estimator, as the data were multivariate and non-normally distributed (Satorra and Bentler, 1994; Brosseau-Liard and Savalei, 2014). Missing data were handled using listwise deletion (Peugh and Enders, 2004). The assessment of model fit was based on the following indicators:(a) chi-square (*χ*^2^); (b) comparative fit index (CFI), best if above 0.90; and (c) root mean squared error of approximation (RMSEA) and standardized root mean squared residual (SRMR), best if below 0.08 (Hu and Bentler, 1999; Kline, 2005; Brosseau-Liard et al., 2012; Little, 2013; Brosseau-Liard and Savalei, 2014).

## Results

### Descriptive Statistics and *t*-Tests

Descriptive analysis and *t*-tests for the main variables are shown in **Table 1**. An independent-samples *t*-test was used to test whether the variables of interest showed significant differences in terms of gender, grade, or area of residence. Furthermore, paired-samples *t*-tests were used to measure the difference between comparison of opinion and comparison of ability and between PSA and NSA. The results revealed that comparison of opinion (*M* = 3.61, *SD* = 0.74) was significantly higher among the participants than comparison of ability [*M* = 2.78, *SD* = 0.67; *t*(1582) = 37.89, *p* < 0.001, *d* = 1.18] and that PSA (*M* = 3.38, *SD* = 0.58) was significantly higher than NSA [*M* = 2.30, *SD* = 0.57; *t*(1301) = 41.36, *p* < 0.001, *d* = 1.88].

**Table 1:** Descriptive statistics and *t*-tests for social comparison orientation, ASC, and social adaptation among Chinese adolescents.

		CO	CA	ASC	PSA	NSA
Gender	Male	3.58 ± 0.78	2.81 ± 0.66	3.80 ± 0.77	3.41 ± 0.60	2.36 ± 0.59
	Female	3.62 ± 0.71	2.74 ± 0.68	3.76 ± 0.70	3.35 ± 0.56	2.25 ± 0.55
	*T*	0.95	2.17^∗^	1.10	2.04^∗^	3.58^∗∗∗^
	*P*	0.342	0.030	0.273	0.042	<0.001
	Cohen’s *d*	−0.05	0.10	0.05	0.10	0.19
Grade	Grade 10	3.64 ± 0.74	2.81 ± 0.67	3.77 ± 0.74	3.36 ± 0.58	2.33 ± 0.57
	Grade 11	3.53 ± 0.74	2.71 ± 0.66	3.79 ± 0.71	3.42 ± 0.58	2.26 ± 0.57
	*T*	2.74^∗∗^	2.64^∗∗^	0.36	2.01^∗^	2.25^∗^
	*P*	0.006	0.008	0.721	0.044	0.025
	Cohen’s *d*	0.15	0.15	−0.03	−0.10	0.12
Area of residence	Rural	3.58 ± 0.73	2.75 ± 0.66	3.72 ± 0.69	3.34 ± 0.57	2.32 ± 0.56
	Urban	3.63 ± 0.76	2.81 ± 0.68	3.85 ± 0.78	3.43 ± 0.59	2.27 ± 0.58
	*T*	1.13	1.81	3.39^∗∗^	2.74^∗∗^	1.70
	*P*	0.261	0.071	0.001	0.006	0.089
	Cohen’s *d*	−0.07	−0.09	−0.18	−0.16	0.09
*M*		3.60	2.78	3.78	3.38	2.30
*SD*		0.74	0.67	0.73	0.58	0.57

*M, mean; SD, standard deviation; CO, comparison of opinion; CA, comparison of ability; ASC, academic self-concept; PSA, positive social adaptation; NSA, negative social adaptation. ^∗^p < 0.05, ^∗∗^p < 0.01, ^∗∗∗^p < 0.001.*

### Correlational Analysis

**Table 2** summarizes the correlations of social comparison orientation, social adaptation, and ASC. Specifically, comparison of opinion was positively related to ASC (*r* = 0.16, *p* < 0.01) and PSA (*r* = 0.29, *p* < 0.01) and negatively related to NSA (*r* = −0.09, *p* < 0.01); furthermore, comparison of ability was positively related to NSA (*r* = 0.22, *p* < 0.01) but not significantly related to PSA (*r* = −0.04, *p* = 0.122) or ASC (*r* = 0.01, *p* = 0.736). In addition, ASC was positively related to PSA (*r* = 0.46, *p* < 0.01) and negatively related to NSA (*r* = −0.31, *p* < 0.01).

**Table 2:** Correlations of social comparison orientation, ASC, and social adaptation among Chinese adolescents.

	1	2	3	4	5
1. CO	1				
2. CA	0.24^∗∗^	1			
3. ASC	0.16^∗∗^	0.01	1		
4. PSA	0.29^∗∗^	−0.04	0.46^∗∗^	1	
5. NSA	−0.09^∗∗^	0.22^∗∗^	−0.31^∗∗^	−0.32^∗∗^	1

*CO, comparison of opinion; CA, comparison of ability; ASC, academic self-concept; PSA, positive social adaptation; NSA, negative social adaptation. ^∗∗^p < 0.01.*

### Measurement and Structural Model

The measurement model consisted of 5 latent factors (PSA, NSA, ASC, comparison of opinion, and comparison of ability) and 18 observed indicators. On the one hand, the measured variables were the four indicators (four dimensions) of PSA, i.e., self-affirmation, pro-social tendency, acting sufficiency, and active coping, and the four indicators (four dimensions) of NSA, i.e., self-trouble, social alienation, violations and social withdrawal. On the other hand, Marsh (1990a) has stated that the general ASC subscale had unidimensionality in its theoretical construction. Accounting for this unidimensionality, the technique of item parceling should be considered (Bandalos and Finney, 2001). Furthermore, Little et al. (2002) stated that random assignment was a simple method of building parcels. Specifically, ASC1 (items 1–4), ASC2 (items 5–7), and ASC3 (items 8–10) on ASC were used.

In addition, social comparison orientation involved comparison of opinion and comparison of ability. Consequently, the initial CFA for the two-factor model showed non-acceptable fit indices: χ^2^(43, 1583) = 545.598, *p* < 0.001; CFI = 0.781; RMSEA = 0.086 (CI = 0.080, 0.092); SRMR = 0.074. The factor loadings of four items (1, 4, 5, and 8) were found to be less than 0.5; therefore, these four items were deleted (Comrey, 1973). After deleting them, the results of the CFA were as follows: χ^2^(13, 1591) = 103.857, *p* < 0.001; CFI = 0.933; RMSEA = 0.066 (CI = 0.055, 0.078); SRMR = 0.042. There were few items on these two single-dimensional variables; therefore, the packaging strategy was not adopted.

**FIGURE 2:**
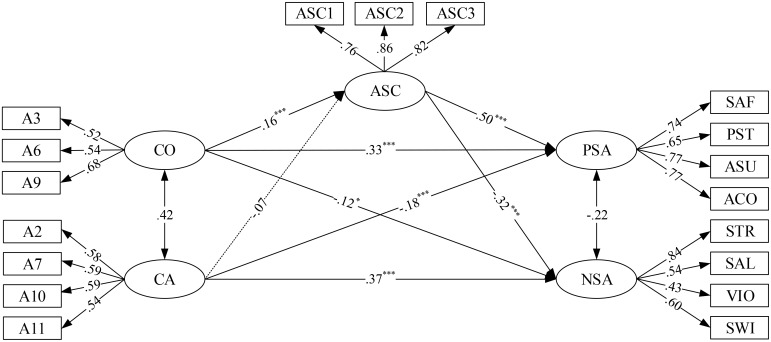
Structural equation model with standardized parameters: social comparison orientation and social adaptation. CO, comparison of opinion; CA, comparison of ability; ASC, academic self-concept; PSA, positive social adaptation; NSA, negative social adaptation; SAF, self-affirmation; PST, pro-social tendency; ASU, acting sufficiency; ACO, active coping; STR, self-trouble; SAL, social alienation; VIO, violations; SWI, social withdrawal; ASC1–ASC3 are three parcels of ASC. A3, A6, and A9 are three items of comparison of opinion; A2, A7, A10, and A11 are four items of comparison of ability; non-significant paths are shown as dotted lines. ^∗^*p* < 0.05 and ^∗∗∗^*p* < 0.001.

The SEM (**Figure 2**) showed a good fit to the data: χ^2^(125, 1202) = 612.159, *p* < 0.001; CFI = 0.911; RMSEA = 0.057 (CI = 0.052, 0.061); SRMR = 0.057. The direct path coefficients from comparison of opinion to PSA (β = 0.33, *p* < 0.001) and NSA (β = −0.12, *p* < 0.05) and the direct path coefficients from comparison of ability to PSA (β = −0.18, *p* < 0.001) and NSA (β = 0.37, *p* < 0.001) were significant. Moreover, the path coefficients from ASC to PSA (β = 0.50, *p* < 0.001) and NSA (β = −0.32, *p* < 0.001) were significant. Furthermore, the path coefficient from comparison of opinion to ASC (β = 0.16, *p* < 0.001) was significant, but the path coefficient from comparison of ability to ASC (β = −0.07, *p* = 0.117) was not significant.

### Mediating Role of ASC

Finally, the tests of indirect effects indicated that ASC had a mediating role in the relationship between comparison of opinion and social adaptation. The indirect effect of ASC between comparison of opinion and PSA was significant (β = 0.08, *p* < 0.001), as was the indirect effect between comparison of opinion and NSA (β = −0.05, *p* < 0.001). Furthermore, ASC did not mediate the relationship between comparison of ability and social adaptation. The indirect effect of ASC between comparison of ability and PSA was not significant (β = −0.04, *p* = 0.112), nor was the indirect effect between comparison of ability and NSA (β = 0.02, *p* = 0.113). **Table 3** presents these results in full.

**Table 3:** Standardized indirect effects from social comparison orientation to social adaptation.

Indirect effect	β (standardized indirect effect)	SE	*P*
From CO to PSA via ASC	0.16 × 0.50 = 0.08	0.022	<0.001
From CO to NSA via ASC	0.16 × (−0.32) = −0.05	0.015	<0.001
From CA to PSA via ASC	(−0.07) × 0.50 = −0.04	0.023	0.112
From CA to NSA via ASC	(−0.07) × (−0.32) = 0.02	0.014	0.113

*SE, standard error; CO, comparison of opinion; CA, comparison of ability; ASC, academic self-concept; PSA, positive social adaptation; NSA, negative social adaptation.*

## Discussion

The present study aimed to test the relationship between social comparison orientation and social adaptation and the mediating role of ASC in this relationship. To the best of our knowledge, this study was the first to unite all these concepts in one testable model. Consistent with the study hypotheses, we found that social comparison orientation was directly and indirectly associated with social adaptation.

### Direct Relations

Social comparison orientation was found to be closely related to social adaptation, and the relationship between the two comparison orientations and social adaptation was in opposite directions. Social adaptation contains cognitive, emotional, and behavioral components (Kahle, 1984). Previous studies revealed that social comparison was related to these three components: cooperative decision making (behavioral component; Gong and Sanfey, 2017), individual life satisfaction (cognitive component; Gibbons and Buunk, 1999), depression, and anxiety (emotional component; Butzer and Kuiper, 2006). Thus, the existence of a direct relationship among social comparison orientation, PSA, and NSA was not surprising. Moreover, the relationship between different comparison orientations and different kinds of social adaptation was inconsistent. This finding further supported the notion that there is a non-social restraint in the process of comparison of ability; however, this restraint is not present in comparison of opinion (Suls and Miller, 1979). When individuals discovered that their opinion was very different from that of others, they could easily change or modify their concept (Suls and Miller, 1979). By sharing each other’s point of view, vocational students may gain conceptual support and better adaptation. Therefore, comparison of opinion was positively related to PSA but negatively related to NSA. Furthermore, when individuals found that their ability was higher than that of others, they did not attempt to change much; however, when individuals found that their ability was lower than that of others, they displayed frustration (Liang and Su, 2015). Previous studies also reported that comparison of ability was negatively related to subjective well-being (Su and Zeng, 2014) and individuals’ self-acceptance (Kim et al., 2017). Therefore, comparison of ability was positively related to NSA but negatively related to PSA. In addition, the score of PSA was significantly higher than that of NSA among the participants in this study, which showed that the social adaptation of vocational students was good. This result was in line with previous studies in China (e.g., Zou et al., 2012).

### Mediated Relations

Our finding argued for the important role of ASC in helping explain the relation between social comparison orientation and social adaptation. More specifically, ASC mediated the relationship between comparison of opinion and social adaptation, while the mediating role of ASC between comparison of ability and social adaptation was not significant in the current study, which roughly supported the study hypotheses.

Previous studies have shown that academic ability was significantly correlated with ASC (Marsh, 1984; Marsh and Parker, 1984). However, it must be emphasized that in this study, ability was not academic ability but general ability, such as social skills and life accomplishment. This comparison of ability was more relevant to an individual’s perception of his/her relative situation in life (Gibbons and Buunk, 1999; Callan et al., 2015). Coincidentally, the results of the paired-samples *t*-test revealed that the score of comparison of opinion was significantly higher than that of comparison of ability, which also further suggested that vocational students paid less attention to comparing abilities than concepts in school. Therefore, the correlation between comparison of ability and ASC was not significant, and the mediating effect of ASC between comparison of ability and social adaptation was also not significant.

As expected, ASC mediated the relationship between comparison of opinion and social adaptation in the current study. This finding linked social comparison theory with the big-fish-little-pond effect; that is, comparison of opinion was not only directly related to social adaptation but also related to social adaptation via ASC. Consistent with these two theories, ASC was rooted in social comparison (Marsh, 1984; Möller and Marsh, 2013) and related to social adaptation (Wouters et al., 2011; Rodríguez et al., 2012; Dadarigashti et al., 2016). For vocational students, acquiring knowledge and opinions is students’ main task during school life. The process of learning or studying is mainly reflected in the acceptance or criticism of others’ views from the perspective of information dissemination (Liang and Su, 2015). Simultaneously, comparison of opinion involves trying to understand oneself and others by comparing similarities, differences, errors, and the merits of ideas (Gibbons and Buunk, 1999). Thus, the processes of studying and comparison of opinion seem to be similar. Therefore, comparison of opinion was correlated to ASC. Regarding the relation between ASC and social adaptation, higher ASC related to more effective social adaptation, such as high educational aspirations (Korhonen et al., 2016), low emotionality (Raufelder and Ringeisen, 2016), and low academic stress (Michie et al., 2001). Therefore, ASC was positively related to PSA and negatively related to NSA. Moreover, this finding further supported the notion that ASC was an intermediate variable between some psychological and educational outcomes (Guo and Bian, 2013). In summary, this finding expanded our understanding that comparison of opinion was related to social adaptation through the mediating role of ASC.

### Implications and Limitations

This study had two outstanding features. From a theoretical perspective, most previous research has mainly focused on the direction of comparison (e.g., upward and downward comparison) and the frequency of comparison (e.g., high comparison frequency). To the best of our knowledge, our findings extended and complemented the knowledge concerning social comparison orientation (e.g., opinion and ability), not only enriching social comparative theory but also showing the differences between comparisons of opinion and ability. From a practical perspective, our findings may help design effective psychological interventions to improve the social adaptation of vocational students. This research highlighted the role of ASC as an intervening variable between social comparison orientation and social adaptation. Therefore, educators should pay more attention to students’ ASC and provide educational programs for the management of ASC. In addition, given the direct relation between social comparison orientation and social adaptation, interventions should develop strategies to make a reasonable comparison. Specifically, educational institutions can encourage students to compare opinions (process) rather than abilities (result) through interventions, which may not only directly improve students’ social adaptation but also improve their ASC as a way to promote social adaptation.

Despite these strengths, this study had certain limitations. First, although we showed a relationship between social comparison orientation and social adaptation, the definition of social adaptation is still general and broad. Therefore, future research should gradually define and unify the concept of social adaptation. Second, we showed the difference between the two comparison orientations. However, we could not fully explain the reasons for such differences; this issue needs to be explored in future research. Third, this study used only self-reported data, which may lead to measurement deviations. In future research, it may be possible to overcome this limitation by measuring students’ social adaptation using teachers’ evaluations. Moreover, there are other internal factors that may affect social adaptation, such as socioeconomic status (Horsburgh et al., 1998; Molyneaux et al., 2016), which could be considered in future studies to test the effect of the independent variables on social adaptation. Finally, testing mediation model with cross-sectional data is not ideal because all variables were measured once. In particular, this study could not show the causal relationships among social comparison orientation (i.e., cause), ASC (i.e., mediator), and social adaptation (i.e., effect); therefore, future longitudinal designs or experimental studies are needed to facilitate causal evaluations.

## Author Contributions

All the coauthors are participants in the data collection and analysis, writing and revising the manuscript.

## Conflict of Interest Statement

The authors declare that the research was conducted in the absence of any commercial or financial relationships that could be construed as a potential conflict of interest.
